# Supplementary material for: Mapping theme trends and recognizing hot spots in postmenopausal osteoporosis research: a bibliometric analysis
Source: PeerJ. 2019 Nov 25;7:e8145. doi: 10.7717/peerj.8145 (PMC6882420; doi:10.7717/peerj.8145)
Supplement: Supplemental Information 1 [file peerj-07-8145-s001.zip › raw data/solution8.html]

## solution 2 - Solution Results

|  |  |  |
| --- | --- | --- |
| **Clustering Options** | | |
| **Method:** Repeated Bisection | | **#Clusters:** 8 |
| **CRfun:** I2 | **Simfun:** Cosine |
| **RowModel:** None | **ColModel:** None | **Graph Model:** Asymetric-Direct |
| **ColPrune:** 1.000 | **EdgePrune:** 0.000 | **VertexPrune:** 0.000 |
| **Nearest Nieghbors:** 4 | **MinComponent:** 1 | **CSType:** Best |
| **#Trials:** 10 | **#Iterations:** 10 |

  
  

|  |  |  |  |  |  |
| --- | --- | --- | --- | --- | --- |
| **8-way clustering:** [36 of 36] | | | | | |
| Cluster | Size | ISim | ISdev | ESim | ESdev |
| 0 | 3 | 0.455 | 0.087 | 0.013 | 0.012 |
| 1 | 3 | 0.447 | 0.092 | 0.013 | 0.005 |
| 2 | 5 | 0.388 | 0.078 | 0.035 | 0.026 |
| 3 | 4 | 0.370 | 0.047 | 0.026 | 0.009 |
| 4 | 5 | 0.302 | 0.035 | 0.021 | 0.008 |
| 5 | 5 | 0.275 | 0.042 | 0.016 | 0.003 |
| 6 | 5 | 0.267 | 0.023 | 0.025 | 0.011 |
| 7 | 6 | 0.263 | 0.024 | 0.025 | 0.015 |

Go to Top  
  

|  |  |  |  |  |  |  |  |  |
| --- | --- | --- | --- | --- | --- | --- | --- | --- |
| **Descriptive & Descriminating Features** | | | | | | | | |
| **Cluster 0**      **Size:** 3     **ISim:** 0.455      **ESim:** 0.013 | | | | | | | | |
| **Descriptive:** | 26108486 | 2.5% | 24162602 | 2.5% | 28419209 | 1.7% | 25138264 | 1.6% |
| **Descriminating:** | 24162602 | 1.3% | 26108486 | 0.9% | 23553199 | 0.9% | 25138264 | 0.9% |
| **Cluster 1**      **Size:** 3     **ISim:** 0.447      **ESim:** 0.013 | | | | | | | | |
| **Descriptive:** | 24698332 | 2.3% | 24712575 | 2.0% | 28748389 | 2.0% | 23824297 | 2.0% |
| **Descriminating:** | 27866217 | 1.1% | 23824297 | 1.1% | 25654380 | 1.1% | 25412945 | 1.1% |
| **Cluster 2**      **Size:** 5     **ISim:** 0.388      **ESim:** 0.035 | | | | | | | | |
| **Descriptive:** | 26661639 | 1.9% | 24619763 | 1.4% | 24269280 | 1.4% | 28131133 | 1.4% |
| **Descriminating:** | 26661639 | 1.2% | 24269280 | 0.9% | 28805099 | 0.9% | 28116510 | 0.9% |
| **Cluster 3**      **Size:** 4     **ISim:** 0.370      **ESim:** 0.026 | | | | | | | | |
| **Descriptive:** | 26642963 | 2.4% | 23436076 | 2.4% | 22572963 | 2.4% | 29114907 | 1.8% |
| **Descriminating:** | 23436076 | 1.4% | 22572963 | 1.4% | 24136103 | 1.1% | 26475289 | 1.1% |
| **Cluster 4**      **Size:** 5     **ISim:** 0.302      **ESim:** 0.021 | | | | | | | | |
| **Descriptive:** | 27072353 | 3.1% | 26121328 | 3.1% | 28013446 | 2.0% | 24386199 | 2.0% |
| **Descriminating:** | 27072353 | 1.8% | 24386199 | 1.2% | 26121328 | 1.1% | 28013446 | 1.0% |
| **Cluster 5**      **Size:** 5     **ISim:** 0.275      **ESim:** 0.016 | | | | | | | | |
| **Descriptive:** | 23680279 | 2.0% | 25288023 | 2.0% | 25373893 | 2.0% | 24281832 | 2.0% |
| **Descriminating:** | 24281832 | 1.2% | 25288023 | 1.2% | 25373893 | 1.2% | 23680279 | 1.2% |
| **Cluster 6**      **Size:** 5     **ISim:** 0.267      **ESim:** 0.025 | | | | | | | | |
| **Descriptive:** | 26234411 | 2.5% | 28613988 | 1.3% | 25708121 | 1.2% | 25722061 | 1.2% |
| **Descriminating:** | 26234411 | 1.6% | 28613988 | 0.6% | 25708121 | 0.6% | 26953481 | 0.5% |
| **Cluster 7**      **Size:** 6     **ISim:** 0.263      **ESim:** 0.025 | | | | | | | | |
| **Descriptive:** | 25857587 | 2.6% | 25734209 | 2.1% | 25314004 | 2.1% | 26760929 | 1.9% |
| **Descriminating:** | 25857587 | 1.6% | 25314004 | 1.3% | 25734209 | 1.3% | 26760929 | 1.2% |

Go to Top  
  
